# Powder Bed Monitoring Using Semantic Image Segmentation to Detect Failures during 3D Metal Printing

**DOI:** 10.3390/s23094183

**Published:** 2023-04-22

**Authors:** Anna-Maria Schmitt, Christian Sauer, Dennis Höfflin, Andreas Schiffler

**Affiliations:** Institute Digital Engineering (IDEE), Technical University of Applied Sciences, Würzburg-Schweinfurt, Ignaz-Schön-Strasse 11, 97421 Schweinfurt, Germany

**Keywords:** additive manufacturing, metal printing, neural network, semantic segmentation, thermal distortion, in situ monitoring

## Abstract

Monitoring the metal Additive Manufacturing (AM) process is an important task within the scope of quality assurance. This article presents a method to gain insights into process quality by comparing the actual and target layers. Images of the powder bed were captured and segmented using an Xception–style neural network to predict the powder and part areas. The segmentation result of every layer is compared to the reference layer regarding the area, centroids, and normalized area difference of each part. To evaluate the method, a print job with three parts was chosen where one of them broke off and another one had thermal deformations. The calculated metrics are useful for detecting if a part is damaged or for identifying thermal distortions. The method introduced by this work can be used to monitor the metal AM process for quality assurance. Due to the limited camera resolutions and inconsistent lighting conditions, the approach has some limitations, which are discussed at the end.

## 1. Introduction

Additive Manufacturing (AM) using Powder Bed Fusion Laser Melting (PBF-LM) is one of the most common technologies to print parts without the need for extensive post-processing steps. The manufactured part is melted out of a metal powder bed. Many studies have been conducted on the properties of the powder bed [1,2,3]. According to [4], the powder bed layer thickness and the recoating step are important factors for the process quality. Derived from these process-critical conditions, quality assurance for additively manufactured parts can be achieved by monitoring the condition of the powder bed and the melted layers. In [5], different monitoring and quality insurance approaches are mapped to the state-of-the-art literature. In addition to others, in situ powder bed monitoring is an approach that is used to track and analyze the properties of a powder bed layerwise during the AM process. This technique allows real-time monitoring of the powder bed and layerwise delayed actions or adaptions, which can be used to improve the accuracy and efficiency of the AM process. By definition, powder bed monitoring is always done layerwise after recoating and processing. The measured values from the powder bed are, in most cases, two-dimensional images, representing the reflected light intensity of the build area at a certain point in time.

The focus of this article is the approach for powder bed monitoring to use a digital camera in visible light wavelength ranges. Using such cameras makes this approach attractive in the sense of costs and fewer integration efforts. The general approach uses layerwise captured images of the powder bed and uses computer vision algorithms to identify, classify, and calculate metrics. This result is then used to trigger actions, such as ‘send warning’ or to generate quality-related documentation for the specific part. Current works in that field use trained neural networks for image processing [6,7] to detect defects or abnormal conditions. The use of neural networks can also support the image analysis process with additional functionalities or with better performances compared to classical unsupervised image processing with edge detection filters or pattern matching [8] to detect the surface deformations of powder-bed images in real time.

The intermediate results presented in this article make use of a novel approach to sequentially combine the use of a trained neural network to segment each image pixel into different classes and classical image processing. Patterns, edges, areas, or area centroids were detected to compare images layerwise with the planned layers. Image segmentation and the use of trained neural networks are common tasks performed in medicine. Brain tumors can be segmented from the background using neural networks [9].

One of the main advantages of powder bed monitoring using camera images is that it allows for the non-destructive analysis of the powder bed. This means that the powder bed can be analyzed without disrupting the AM process or damaging the powder.

### Related Works

Most of the known approaches for powder bed monitoring focus on detecting defects in the recoating process. Approaches based on images of the whole build platform can be categorized into visible wavelength and infrared images. Using visible wavelength images, approaches can be divided into before and after recoating images.

An approach before recoating was described in [8]. The authors used automated anomaly detection for computer tomographic scans to label discontinuities. These 3D-labeled voxels were transferred into 2D labels for the layerwise images. Support Vector Machines (SVMs) were trained to classify different features into the classes, namely ‘defect’ and ‘ok’. Using these SVMs, an ensemble classifier achieves an accuracy of 85%. Pagani et al. [10] extracted the contour of the parts after a preprocessing step and defined a metric to compare the actual contour to the reference contour. Different contour segmentation algorithms are also discussed in [11] with different preprocessing and lighting conditions. Reference [12] describes a procedure to transform the image pixels as graph vertices and segment the image by using the min-cut max-flow algorithm.

Six different defects and one ‘ok’ class after recoating are classified in [13]. The authors used the built-in powder bed camera, preprocessed the captured images, and used patches as input for the bag-of-words machine learning technique. Similar defects were detected by Fischer et al. [4]. Using an Xception neural network architecture extended with a fully connected layer, 9 classes (8 defects + 1 ‘ok’) were detected. The used images were different-sized patches with different lighting conditions. In the experiments, the largest patch sizes of 6 mm × 6 mm with the highest resolution of 6 μm/px and dark field lighting led to the highest accuracy of 99.15%.

Baumgartl et al. [14] used thermographic images with a resolution of 224 × 224 × 3 px and a Convolutional Neural Network (CNN) to classify delamination, splatters, and no defects. In [15], the authors used images from different machines for their approach. The input of the dynamic segmentation CNN consisted of four images (infrared spreading, visible spreading, infrared fusion, and visible fusion). The architecture had three different parts considering the local (pixel-wise classification), regional (U-Net), and global (CNN) information, and classified every pixel into powder, part, and different defects. The model was trained with the high effort needed due to manually labeled images.

## 2. Research Target

Thus far, no approach has used the given desired layered part section to compare it with the melted and captured layers using digital cameras in the range of visible light wavelengths. This research employs a trained neural network to classify each pixel and perform a segmentation of melted and unmelted areas. The neural network is used to achieve better segmentation results compared to classical non-parametric unsupervised algorithms such as Otsu’s segmentation algorithm. The needed annotated images or masks for the neural network training are automatically created using the sliced layer data from the desired part. The presented approach uses semantic segmentation on the whole image to pixel-wise classify powder and part, in contrast to Fischer et al. [4], who use patches. Therefore, with this approach, no training data with defects and no manual annotation are required.

After segmentation, a predicted mask is available to which various filters or calculations can be applied. For example, the size of melted areas, area centroids, and logical operations of the predicted melted area from captured powder bed images and target melted areas from slicing the part can be calculated.

The research goal is to prove the concept of comparing image masks of melted areas, one derived from slicing and the other from the powder bed image, and to calculate valuable metrics for quality monitoring. To achieve this, a low-cost camera with low resolution (1280 × 1024 px) was chosen, which achieves a powder bed resolution of 300 μm/px and captures single-shot images per melted layer.

## 3. Materials and Methods

**Figure 1:**
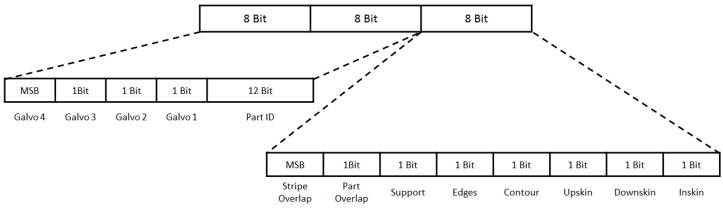
Bit encoding for each pixel in the layerwise planned slice of the parts.

The research was conducted in the additive metal printing research lab at the Technical University of Applied Sciences Würzburg-Schweinfurt, where a 3D printer (EOS M290) is available and stores powder bed images from build jobs in a database. The grain size of the metal powder typically used ranges from 10 to 25 μm, and the maximum part size is within a cube with an edge length of 250 mm. The approach focuses on using the black and white camera images from the built-in device, which captures images with a resolution of 1280 × 1024 px and a field of view of the whole powder bed. The camera is connected via USB and the exposure is triggered every time the melting of a layer has finished. Geometrical corrections and cropping were performed to select the building platform as the region of interest. One advantage of the built-in equipment and stored images is that, for each printed layer, a reference image is available that contains different information encoded in a specific scheme. Every pixel of this single-channel mask has 24 bits, which encode information about the desired part process features shown in Figure 1 as a reference (Note: To access this information, a specific license is needed.).

To use this mask for model training and evaluation, only the part identification (12 bits) and the last 5 bits are used to identify the desired melted area of the part. The created mask was stored as a PNG file. All pixels with a value of >0 belong to the melted area (part), and each pixel with a value of 0 is considered powder. A pixel value of >0 represents the part identification.

**Figure 2:**
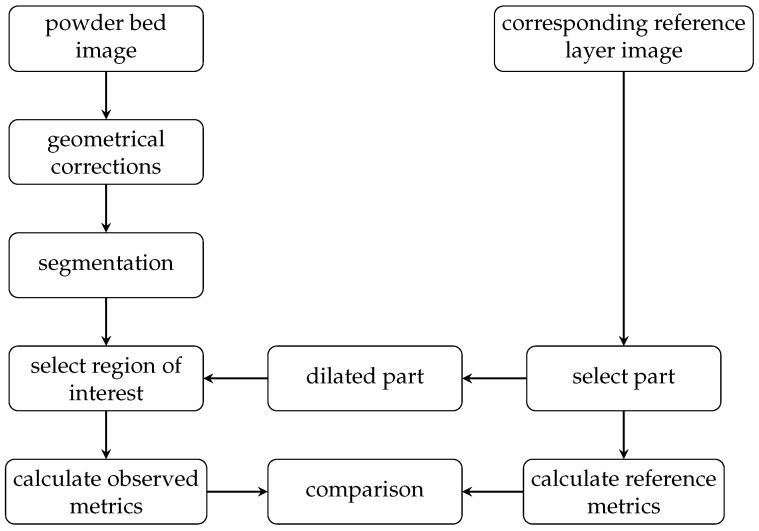
Flowchart of the monitoring approach.

The basic idea of the monitoring approach is not to detect or classify defects. Instead, it is about detecting deviations from the planned process and, therefore, being able to set thresholds on geometrical metrics, such as positions, areas, and area centroids. This approach requires a reference layer with at least the part identification and the location of the melted areas (pixels). This information is provided as described earlier. On the other hand, an observed layer is needed, which represents the actually produced layer. This actual layer is calculated based on an image captured by the powder bed camera. A flowchart for the monitoring approach is shown in Figure 2.

### 3.1. Monitoring Approach

The major challenge is to create an image segmentation that classifies each captured pixel of the powder bed into at least two classes. Class 1 is the melted area, and class 0 is the metal powder (background). The second piece of information after classification is an annotation of each pixel classified as a melted area with the corresponding part ID from the reference layer. This step is performed with a dilated part mask created using the reference layer.

In the next sections, the segmentation steps (pixel-wise classification), part selection (annotated classified pixels with a part id), and calculation of 2D monitoring metrics are described in detail.

### 3.2. Segmentation Approach

Classifying each pixel of the powder bed image into the melted area and background (metal powder) is challenging due to the following conditions:

- The reflected light intensity is not homogeneous over the powder bed.
- Due to the surface structure—stripes—of the melted metal, the light reflections have different orientations.
- The heat distribution on the surface is not homogenous, although the camera has a low-pass filter, the measured intensity still results in biased brightness values due to the surface temperature.

Imani et al. [16] describe that in optical images, the contrast between the powder and part is low, leading to problems in defect detection with neural networks. On the one hand, non-parametric, unsupervised approaches, such as static thresholds, adaptive thresholds, or algorithms such as the Otsu method (known from publications in the early 1980s), can be used. Such algorithms are available and implemented in the well-known software library OpenCV. On the other hand, there are model-based and supervised approaches, such as CNNs or SVMs. The advantage of such methods is that they can learn from data and are, therefore, able to adapt to different conditions. The disadvantage is that they require a lot of training data and more computing power.

**Figure 3:**
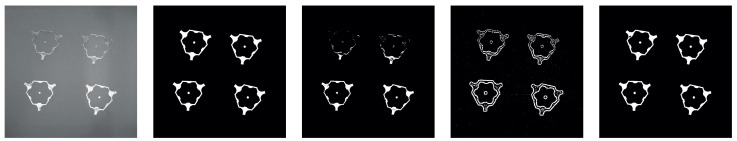
Comparison of different algorithms for segmentation. **Left**: input image, **Mid-Left**: mask derived from reference layer information, **Mid**: OpenCV Otsu’s thresholding, **Mid-Right**: OpenCV adaptive threshold, **Right**: segmentation using the specifically trained Xception model.

The straightforward non-parametric methods for segmentation are not sufficient in their results. The tests and applications of such methods were carried out on images where the melted areas perfectly matched the reference layers. A solution using only OpenCV-provided methods was tested but proved to be insufficient. In Figure 3, an image of an example layer is displayed on the left. The second image from the left is the corresponding mask calculated from the reference image. In the middle and second-to-right pictures, the segmentations using the OpenCV Otsu’s and adaptive thresholding methods are displayed, respectively. In comparison, the rightmost image shows the segmentation using a specifically trained Xception neural network.

Thus, the more promising approach is to train a model with reference data to be able to classify each pixel in the captured powder bed image. As the network architecture, a U-Net shaped model with a convolutional layer was chosen. This architecture is known and described in its use for automated medical image analysis [17]. Once the CNN is trained, it can be applied to new images to generate a “segmentation map” that shows the predicted class for each pixel. Using a neural network to predict the class of each pixel in a captured image is a supervised approach. This results in the need for pixel-wise annotated training data. In the work presented by Scime et al. [15], manual annotation is used. This means there was a great effort to annotate a set of at least 2000 images for different areas and lighting conditions.

The approach focused on here uses reference images for each layer, which are part of the build job files. Therefore, it is not necessary to train on defects, and the required pixel-wise labeling can be derived directly from the reference layers. Additionally, images of ‘build jobs’ without defects and with diverse light and reflection conditions can be used. The training data were derived from six different build jobs, which were built without issues.

The training was conducted in three steps:

1. Obtaining the powder bed and reference layer images from the database.
2. Extracting the corresponding mask for training.
3. Training the neural network with actual images as input and the reference masks as targets.

In the first step, images with a resolution of 1280 × 1024 px were taken after laser exposure for every layer, saved in a database, and extracted after the build job was finished. The images were then cropped and geometrically corrected to 750 × 750 px. The next step is to process the reference layers, which encode a variety of information as shown in Figure 1. To label these images, only pixels with a value of 1 in one of the last 5 bits and a 0 in the bits 13 to 15 are kept as 1 (part), while the rest are labeled as 0 (powder). The original resolution of the stored reference layers is 2000 × 2000 px, but they are downscaled to match the layer image resolution of 750 × 750 px after processing, and this downscaled version is considered the annotated label.

**Figure 4:**
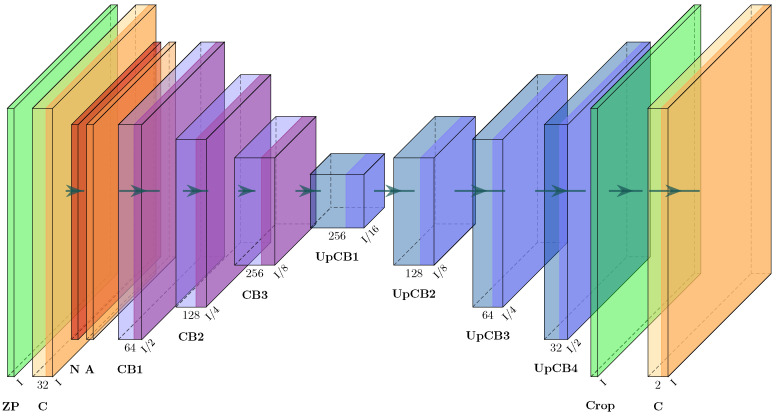
Modified Xception architecture derived from [18].

The final step is training the neural network, originally described in [17] and extended in [18] (see Figure 4 and Appendix A). The resolution of 750 × 750 px can only be halved once until the resolution is an odd number and cannot be halved again. Therefore, the first layer, zero padding, is added to obtain a resolution of 752 × 752 px. In the last layer, these zeros are cropped to match the target resolution.

The neural network was trained for approximately four hours on two Nvidia RTX A5000 graphic cards with a batch size of 20 on 8959 images taken from 6 different print jobs. As metrics to evaluate the neural network on 2240 random test images, the accuracy and the mean Intersection Over Union (IoU), also known as the Jaccard Index, were used. The mean IoU for object detection was calculated by dividing the intersection of prediction and ground truth by the union of both sets for every available class. If the images have an imbalanced class distribution (e.g., 95% background), only using accuracy as a metric is insufficient as the model would achieve an accuracy of 95% by solely classifying the background. The mean IoU penalizes this misclassification [19]. After training for 15 epochs, a test accuracy of 95.7% and a mean IoU of 99.1% were achieved.

Finally, the trained model predicts two classes for each input image pixel: Class 1 represents melted areas and class 0 represents metal powder (background). The output of this segmentation is referred to as the observed layer image, in contrast to the reference layer image.

### 3.3. Annotating the Predicted Segmentation with the Part Identification

The number of pixels belonging to a part can change drastically depending on part geometry. In other research articles, the Computer-Aided Design (CAD) file of the part was used to extract the regions of interest [16]. The region of interest in this research is determined using the reference layer information.

**Figure 5:**
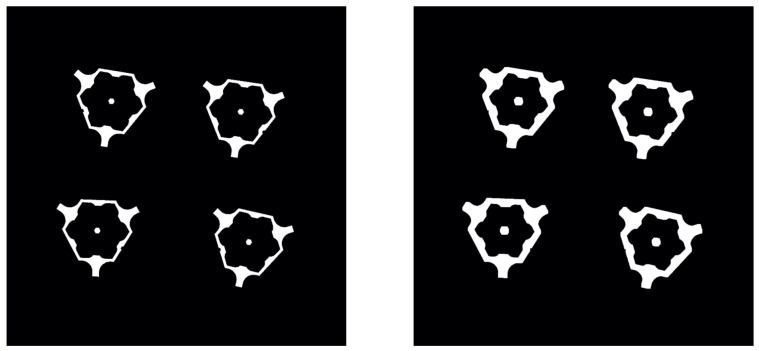
Reference layer (**left**) and dilated reference layer (**right**); showing four parts.

If a print job has multiple components (parts), the corresponding region in the image has to be determined. The pixels belonging to one part in the reference layer can be derived from bits 5 to 16 (see Figure 1) and are set to 1, while all other pixels are set to 0. To extract the corresponding pixels from the segmentation image, the areas from the reference layer are dilated with a 7 × 7 kernel (see Figure 5) [20]. This approach assumes that pixels classified as melted metal with a tolerance of 3 pixels belong to the same part. With the given resolution of 3 pixels per millimeter, this assumption means that pixels classified as melted metal belong to the same part with a maximum tolerance of one millimeter. In the observed layer image, only the pixels that are also 1 in the dilated reference layer are kept as 1. Using a rectangular bounding box to identify the part is not sufficient because the box can intersect with other parts on the building plane. Therefore, annotating the pixels classified as melted metal with the correct corresponding part identification is necessary to use the predicted melted part in subsequent calculations.

### 3.4. Metrics

At this point in the monitoring procedure, a reference layer image and an observed layer image segmented by a trained neural network were generated. The possible operations that can be applied to the two images can be manifold to monitor deviations from the planned layers in the print job. The metrics chosen and tested in this work are:

- Area deviation.
- Distance between the area centroids.
- Normalized area difference of an image generated by applying the logical operation *xor* on both images.

The area of every part *k* in a layer $a_{L}$ was calculated by using the count of the pixels *p* classified as the part (see Equation (1) with *N* as the total number of pixels).

(1) $$a_{k,L}=\sum_{i=1}^{N}p_{i}\;\forall p_{i}=\left\{\begin{array}{cc} 1 & \mathrm{if}\;\mathrm{the}\;\mathrm{pixel}\;\mathrm{is}\;\mathrm{classified}\;\mathrm{as}\;\mathrm{part}\;k\\ 0 & \mathrm{else} \end{array}\right.$$

Using the observed layer image from Section 3.3, the area of the part can be calculated using Equation (1). However, actual and target areas may have a systematic deviation due to imperfect optical corrections for the geometrical distortions. To compensate for this systematic bias, the deviation factor of the first 10 layers is determined and then multiplied to all predicted values. This calculation assumes that at the beginning of the print job, the first melted layers will perfectly match the reference layers because thermal distortions or other process defects will not occur at the very beginning.

Calculating the area of each part and comparing it to the reference will support identifying massive defects during the print job, for example, if regions of a part abort due to recoater collisions.

The use of the calculated area centroid will support finding slow-growing thermally inducted geometrical distortions. The centroid coordinates $c_{L}$ for each part are determined by the sum of all coordinates in *x*- and *y*-directions, respectively, and divided by the area (see Equations (2a) and (2b)).

(2a) $$c_{x,L}=\frac{1}{a_{L}}\cdot \sum_{i=1}^{N}p_{i,x}$$

(2b) $$c_{y,L}=\frac{1}{a_{L}}\cdot \sum_{i=1}^{N}p_{i,y}$$

As a metric that can be observed for print job monitoring, the Euclidean distance between the predicted centroid and the reference centroid is used. This metric is calculated for every part.

The third metric used represents the ratio of the area of the difference between the target and actual image to the target area. To calculate the area of difference, the actual pixel values $p^{a}$ and the target pixel values $p^{t}$, which are solely 1 and 0, are used in the exclusive or function (see Equation (3a)). Therefore, only pixels that have different values in both images are counted. The metric is then normalized with the target area of each layer per part $a_{L}^{t}$ (see Equation (3b)).

(3a) $$a_{d,L}=\sum_{i=1}^{N}p_{i}^{a}⊕p_{i}^{t}$$

(3b) $$a_{d,L,\mathrm{norm}}=\frac{a_{L}^{t}-a_{d,L}}{a_{L}^{t}}$$

At this point, three different metrics have been defined. The calculated results provide a single value per part for each layer, and these values can be monitored using simple thresholds across all printed layers. It cannot prevent defects, but it can support early detection.

### 3.5. Proof of Concept

**Figure 6:**
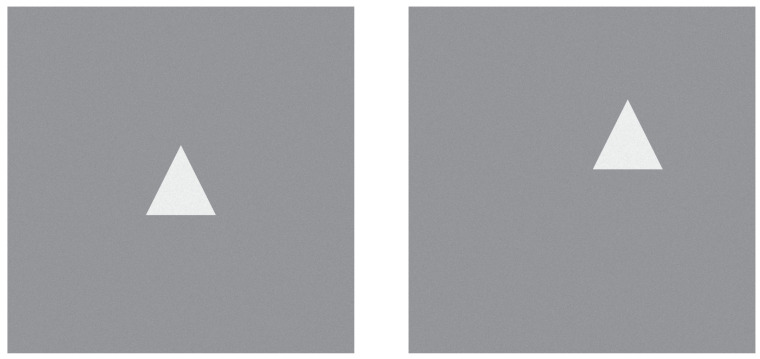
First test image (**left**) and 100th test image (**right**).

Test cases were created to verify the calculations using reference layer images compared to observed layer images. The test case consists of a single triangular part with a fixed size and, therefore, a fixed area. The position of the part changes layer by layer; 100 test images were created with a centroid shift of 1 pixel per image, and a gray value of 128 for the background (metal powder) and 235 for the melted metal area. To simulate disturbances, a Gaussian distribution was added to every pixel. Figure 6 shows two examples of the generated test images.

**Figure 7:**
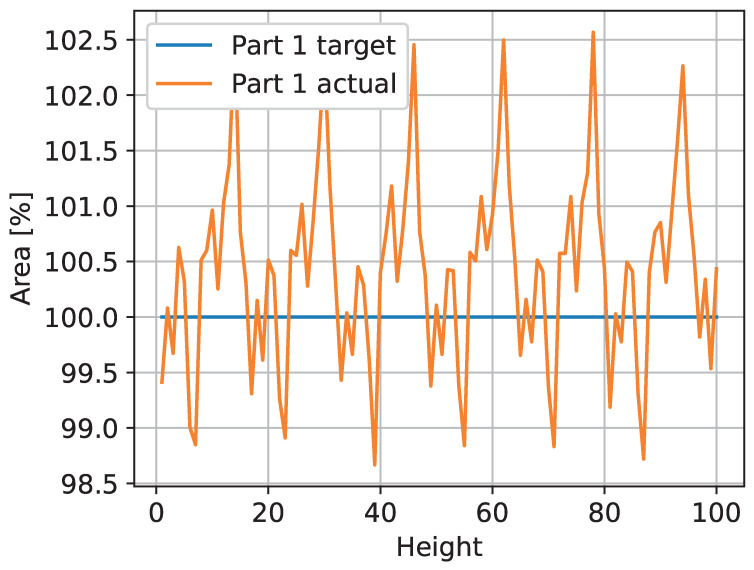
The area of the ideal triangle cannot be represented exactly by the given resolution. Moving the triangle along a diagonal line results in a periodic count of the pixel-based area.

The trained neural network was applied to the generated test images to create the observed layer images. Metrics were calculated for these images. In Figure 7, the areas of the 100 test images are shown. The target area is 100% pixels for every layer, and the predicted area deviates between 98.7% and 102.6% after normalization.

**Figure 8:**
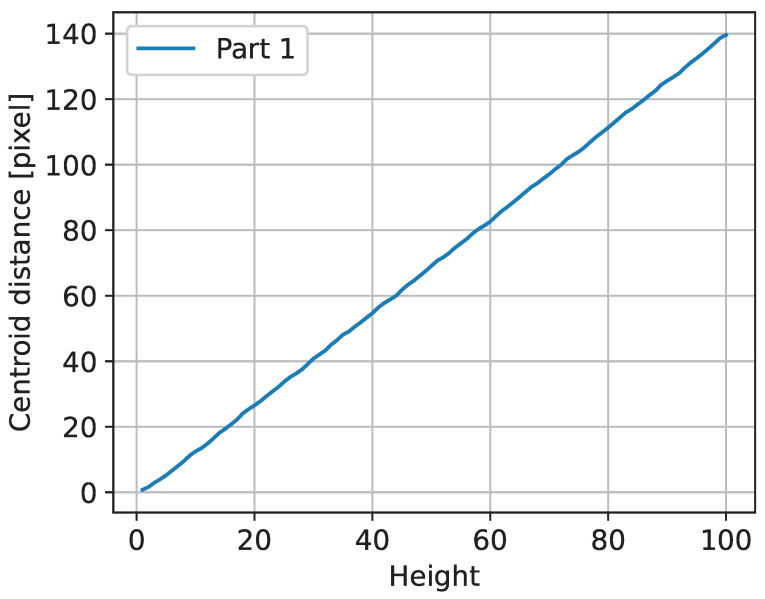
Plot of the triangle centroid distances.

Figure 8 shows the calculated distance in pixels between the predicted and actual centroids of the triangles. The results of the test case show that the implemented algorithms are correct in principle, and that for the given example, the observed distance between the area centroids can be used to monitor deviations.

## 4. Results

**Figure 9:**
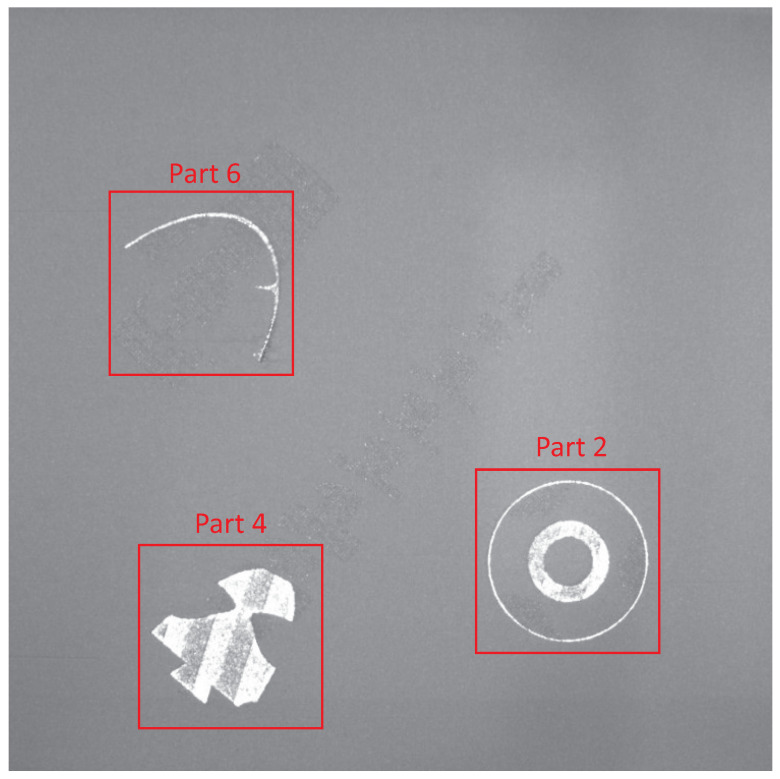
Example powder bed image with marked parts at a height of 20 mm from the evaluated build job.

To evaluate the approach on real production data, one print job with a defect and thin-walled parts was chosen. The job consisted of three different parts, as shown with their identification numbers in Figure 9. This print job was selected to test the limits of the approach. The challenges were the thin structures with widths of less than one millimeter and solid parts with conspicuous reflections due to melted strips. At a certain level, part 6 failed by a thermal deformation and collision with the recoater. The further processing of that part was stopped (refer to Figure 9). The whole job consisted of 3459 layers and images, resulting in a maximum height of 106 mm.

All results and calculated metrics are given in millimeters instead of pixels. The image resolution in the given setup is $300\frac{\mu m}{\mathrm{pixel}}$. This is a lower resolution compared to the setup used by the authors of [21], where the approximate pixel size of the camera system was $200\frac{\mu m}{\mathrm{pixel}}$.

**Figure 10:**
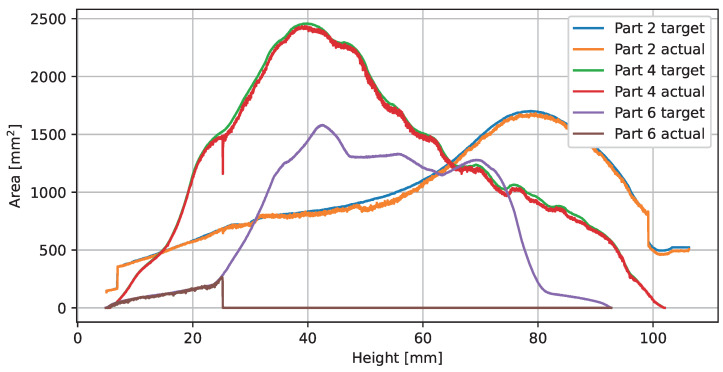
Plot of the print job areas.

Figure 10 shows the plot of the reference and observed part areas over the building height. Parts 2 and 4 were printed correctly and without defects, according to expert inspection at the end of the print job. Part 6 was critical due to the thin structure. At a print height of 25.1 mm, a deformation and collision with the recoater occurred, which led to the exclusion of the part from the process. The print job was paused at this point and resumed after several minutes. Overall, Figure 10 does not provide much additional valuable information. It should be noted that the observed layer images are not valid until the 5 mm height base support is finished.

**Figure 11:**
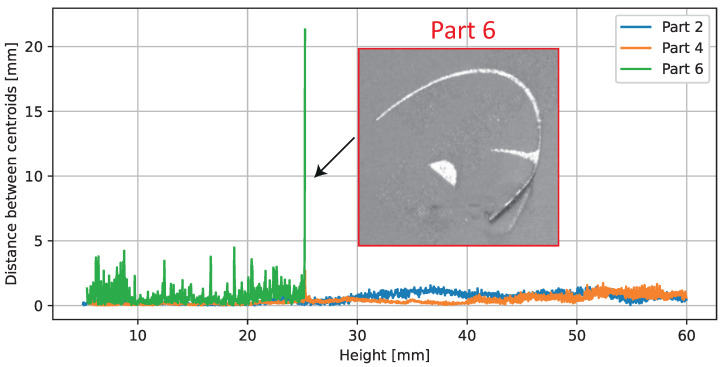
Calculated observed distance for the area centroids over the building height. On height 25.1 mm, a collision occurred between part 6 and the recoater. Upon further processing, part 6 was excluded.

Figure 11 shows the distances between the area centroids of the parts. The centroid distances for parts 2 and 4 are below 1.5 mm for all layers. The observed distances in the area centroids for part 6 show several peaks and a higher standard deviation compared to the other parts. The highest observable peak with distances of more than 5 mm is located at the building level after part 6 was damaged due to a collision with a recoater.

**Figure 12:**
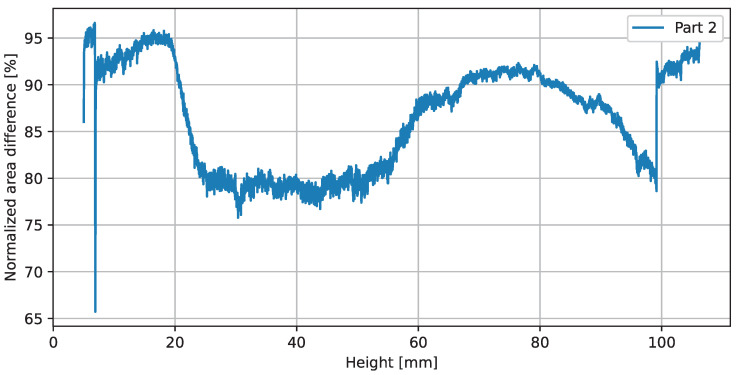
Plot of the normalized area differences.

**Figure 13:**
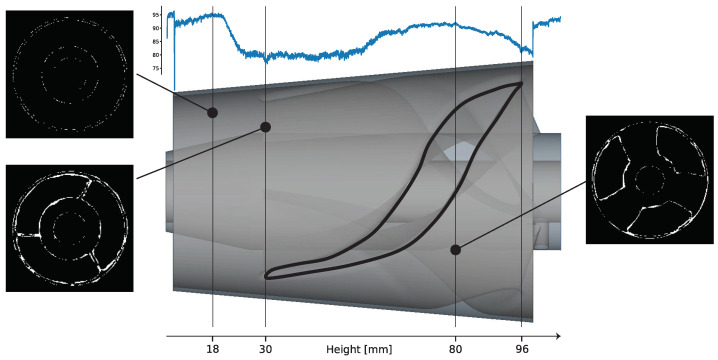
Normalized area differences over the 3D model. The image on the top left shows the xor image of height 18 mm. Bottom left shows the xor image of height 31 mm and the right image shows the xor image of height 80.8 mm.

A side view of part 2 is shown in the middle of Figure 12. It consists of two thin-walled concentric hollow cones, which are interconnected in the build-height range from 30 to 96 mm by a screwed impeller structure. In addition to the shown CAD representation of the part at the top of Figure 13, the normalized area difference (refer to Section 3.4) is plotted over the building height. At three specific positions, images generated with the xor operation on the observed layer image and the reference layer image are shown to illustrate the deviations. An ideal congruent layer image would have a value of 100% match, indicating that no pixel differs between the reference layer image and the observed layer image. Thus, any value below 100% indicates a difference or misclassified pixels. Comparing this result with the geometrical definition of the manufactured part suggests that there is a thermal deformation in the build height region (30 to 96 mm) where the concentric cylinders are connected by solid metal with a varying cross-sectional area (refer to the vertical lines in Figure 13). Based on this calculated metric, it is not possible to directly derive the absolute value of the deviation. Therefore, a supervised set threshold is required to define a valid range. Investigations to compare the graph with tactile-measured geometrical deviations have not yet been performed and will be part of future work.

## 5. Discussion

One common cause of failure of parts considered in this research is overheating. As thermal distortion cannot be corrected, it is essential to detect and intervene. This creates the need to develop a control based on the evaluated metrics (see Section 6.2).

The chosen print job highlighted several limitations of the described methods. One of them is the relationship between the dimensions of thin parts and the theoretical image resolution. The thin-walled structure of part 6 and the outer circle of part 2 are examples of this. The smallest part thickness is between 1 and 2 mm, resulting in only 3–5 pixels in width, which can lead to incorrect segmentation or less precise position results. Using a camera with a higher resolution could be a solution to this. Alternatively, another image-capturing device, such as a line camera attached to the recoating device, could also increase resolution and precision. In addition, retraining the neural network with new images, especially those with challenging conditions for segmentation, would be a promising next step.

**Figure 14:**
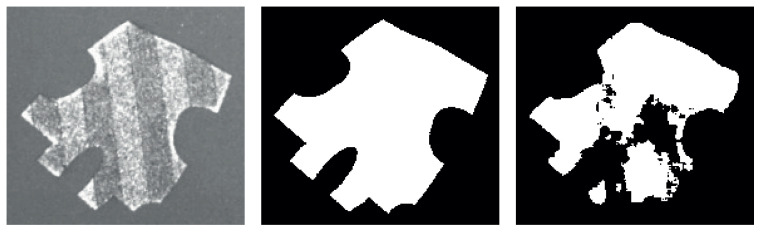
Segmentation error of part 4 at height 25.2 mm due to inhomogeneous lighting. Left: Camera image, middle: mask, right: segmentation.

The limitation and drawback of the presented approach is the need for images of the reference layer. This results in the necessity of either creating them from the 3D part files or using given software functionalities to extract them from databases. Moreover, inhomogeneous lighting conditions will result in errors in image segmentation. Figure 14 shows part 4 at a height of 25.2 mm, where dark reflections are visible, leading to misclassifications because they are close to the gray values of the nearby unmelted powder. One solution known from the state-of-the-art would be to use multi-exposure techniques with varying lighting directions. For instance, Abdelrahman et al. [22] used up to five images taken with different light sources.

## 6. Conclusions and Further Research

### 6.1. Conclusion and Outlook

In the presented work, a neural network was chosen to segment between the melted part and the powder. After training, an accuracy of 95.7% and a mean IoU of 99.1% were achieved. During the evaluation, some limitations, such as inconsistent lighting and low camera resolution, were identified to negatively affect the segmentation results.

The chosen metrics are generally suitable for evaluation. The observed area can be used in the print job documentation. If a part is ripped from the rest by the recoater, the distances between the centroids and the normalized area differences are valuable metrics. Nonetheless, further metrics can be defined and evaluated.

Another limitation is the high segmentation error of a small melted area. This can be avoided by starting the evaluation at a higher layer. Additionally, retraining with smaller melted areas and finer part structures could increase the segmentation.

In this research, the print job evaluation was performed after its completion. For in situ monitoring, the powder bed image and the corresponding reference layer image must be available directly after the layer is finished printing. Using one Nvidia A5000 graphics card, segmenting a 750 × 750 px image takes around 100 ms. This is essential for an in situ monitoring approach, as every layer image can be evaluated before the new image is captured. Using this approach on an embedded device or PLC increases the need for computing acceleration (Note: e.g., Google TPU, Hailo H8) to ensure deterministic behavior, which is mandatory in a productive use case.

Due to the automatic labeling process presented in this research, every layer of a successful print job can be used for (re)training. Therefore, the training data are not limited as it is in other research. This neural network was trained with 8959 images, compared to the approach in [15], which used 94-layer images.

### 6.2. Further Research

The architecture and hyperparameters of the neural network used were not optimized in this research. Therefore, different architectures will be compared in future research. Additionally, the optimal hyperparameters will be searched to improve the segmentation results.

The class distribution of powder and part is highly imbalanced, as the number of powder pixels exceeds in every image. A method to consider for future model training runs is to use different loss functions. The focal loss function was defined to reward harder-to-classify classes by [23]. Another approach is to specify different class weights for the cross-entropy loss function [24]. These two adjusted approaches for training will be part of further research.

Each print job automatically generates images that are available as training images. Retraining the model after every job with the new images and the influence on the evaluation will be further investigated. This could enhance the segmentation results of the neural network and ensure that more images of different lighting conditions and fine-structured parts are considered. Additionally, a higher image resolution as well as multiple light sources with different directions are considered.

## Figures and Tables

**Figure A1:**
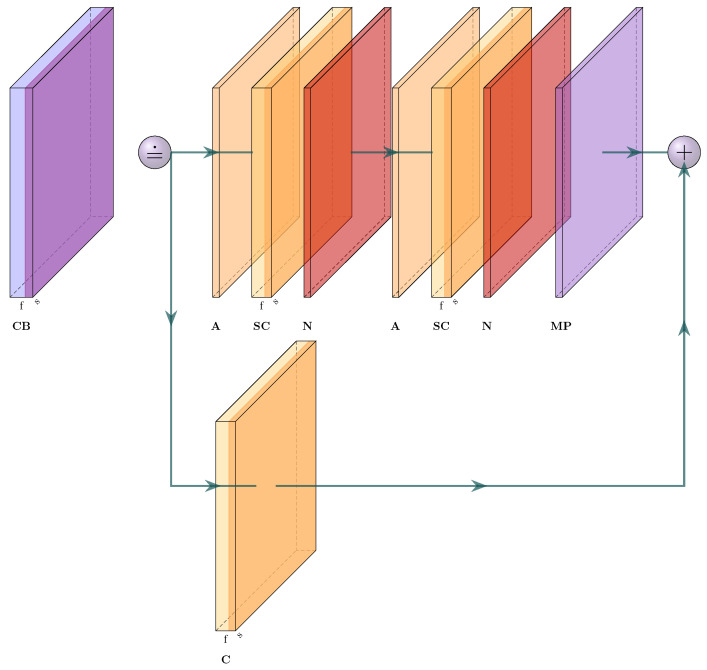
Convolutional block.

**Figure A2:**
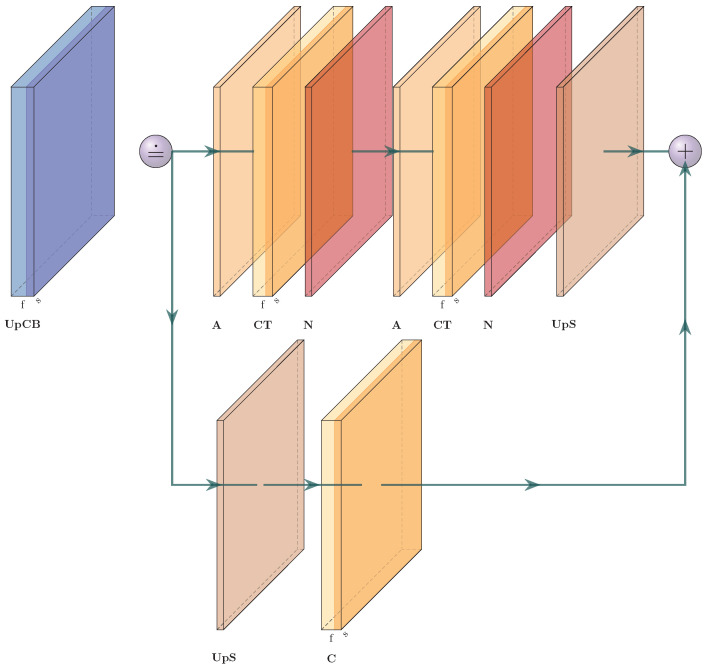
Upsampling convolutional block.

## Data Availability

A total of 132 images and their corresponding reference layer images were uploaded to a repository. Additionally, the source codes for training the neural network and evaluating the images were added to the repository. They are available at https://github.com/SuperAms/Powder-Bed-Monitoring-Using-Semantic-Image-Segmentation (accessed on 10 March 2023).

## Abbreviations

The following abbreviations are used in this manuscript:

AM	Additive Manufacturing
CAD	Computer-Aided Design
CNN	Convolutional Neural Network
IoU	Intersection Over Union
PBF-LM	Powder Bed Fusion Laser Melting
SVMs	Support Vector Machines

## Appendix A. Xception Architecture

The Xception architecture consists of several convolutional blocks. To enhance readability the architecture shown in Figure 4 was simplified. The convolutional blocks (CB) and upsampling convolutional blocks (UpCB) are shown in Figure A1 and Figure A2. The abbreviations for the layers used in Figure 4, Figure A1 and Figure A2 are listed in Table A1.

**Table A1:** Description of used layers.

Abbr.	Layer	Description
A	Activation layer	The used function is ReLu.
C	Convolutional layer	The number of used filters is at the bottom mid.
		The input image size is in the lower right corner.
CB	Convolutional block	The block consists of several layers.
Crop	Cropping (2D)	Delete the edge pixels to match the desired output
		resolution.
CT	Transposed convolutional layer	With f as the number of filters and s as the size.
MP	MaxPooling	Decrease image resolution.
N	Batch normalization	Standardize inputs.
SC	Seperable convolutional layer	With f as the number of filters and s as the size.
UpCB	Upsampling convolutional block	It consists of several layers.
UpS	Upsampling layer	Increase image dimensions.
ZP	Zero padding (2D)	Add 1 pixel at the edges.
		The result is an image with size *I* (lower right corner).

## References

[1] Vock S., Klöden B., Kirchner A., Weißgärber T., Kieback B. (2019). Powders for powder bed fusion: A review. Prog. Addit. Manuf..

[2] Slotwinski J.A., Garboczi E.J., Stutzman P.E., Ferraris C.F., Watson S.S., Peltz M.A. (2014). Characterization of metal powders used for additive manufacturing. J. Res. Natl. Inst. Stand. Technol..

[3] Strondl A., Lyckfeldt O., Brodin H., Ackelid U. (2015). Characterization and control of powder properties for additive manufacturing. Jom.

[4] Fischer F.G., Zimmermann M.G., Praetzsch N., Knaak C. (2022). Monitoring of the Powder Bed Quality in Metal Additive Manufacturing Using Deep Transfer Learning. Mater. Des..

[5] Grasso M., Remani A., Dickins A., Colosimo B.M., Leach R.K. (2021). In-Situ Measurement and Monitoring Methods for Metal Powder Bed Fusion: An Updated Review. Meas. Sci. Technol..

[6] Foster B.K., Reutzel E.W., Nassar A.R., Hall B.T., Brown S.W., Dickman C.J. Optical, Layerwise Monitoring of Powder Bed Fusion. Proceedings of the 2015 International Solid Freeform Fabrication Symposium.

[7] Ansari M.A., Crampton A., Parkinson S. (2022). A Layer-Wise Surface Deformation Defect Detection by Convolutional Neural Networks in Laser Powder-Bed Fusion Images. Materials.

[8] Gobert C., Reutzel E.W., Petrich J., Nassar A.R., Phoha S. (2018). Application of Supervised Machine Learning for Defect Detection during Metallic Powder Bed Fusion Additive Manufacturing Using High Resolution Imaging. Addit. Manuf..

[9] Ben naceur M., Akil M., Saouli R., Kachouri R. (2020). Fully Automatic Brain Tumor Segmentation with Deep Learning-Based Selective Attention Using Overlapping Patches and Multi-Class Weighted Cross-Entropy. Med. Image Anal..

[10] Pagani L., Grasso M., Scott P.J., Colosimo B.M. (2020). Automated Layerwise Detection of Geometrical Distortions in Laser Powder Bed Fusion. Addit. Manuf..

[11] Caltanissetta F., Grasso M., Petrò S., Colosimo B.M. (2018). Characterization of In-Situ Measurements Based on Layerwise Imaging in Laser Powder Bed Fusion. Addit. Manuf..

[12] zur Jacobsmühlen J., Achterhold J., Kleszczynski S., Witt G., Merhof D. (2019). In Situ Measurement of Part Geometries in Layer Images from Laser Beam Melting Processes. Prog. Addit. Manuf..

[13] Scime L., Beuth J. (2018). Anomaly Detection and Classification in a Laser Powder Bed Additive Manufacturing Process Using a Trained Computer Vision Algorithm. Addit. Manuf..

[14] Baumgartl H., Tomas J., Buettner R., Merkel M. (2020). A Deep Learning-Based Model for Defect Detection in Laser-Powder Bed Fusion Using in-Situ Thermographic Monitoring. Prog. Addit. Manuf..

[15] Scime L., Siddel D., Baird S., Paquit V. (2020). Layer-Wise Anomaly Detection and Classification for Powder Bed Additive Manufacturing Processes: A Machine-Agnostic Algorithm for Real-Time Pixel-Wise Semantic Segmentation. Addit. Manuf..

[16] Imani F., Chen R., Diewald E., Reutzel E., Yang H. (2019). Deep learning of variant geometry in layerwise imaging profiles for additive manufacturing quality control. J. Manuf. Sci. Eng..

[17] Ronneberger O., Fischer P., Brox T. U-net: Convolutional networks for biomedical image segmentation. Proceedings of the Medical Image Computing and Computer-Assisted Intervention–MICCAI 2015: 18th International Conference.

[18] Chollet F. Xception: Deep learning with depthwise separable convolutions. Proceedings of the IEEE Conference on Computer Vision and Pattern Recognition.

[19] Rahman M.A., Wang Y. Optimizing Intersection-Over-Union in Deep Neural Networks for Image Segmentation. Proceedings of the Advances in Visual Computing.

[20] Haralick R.M., Sternberg S.R., Zhuang X. (1987). Image Analysis Using Mathematical Morphology. IEEE Trans. Pattern Anal. Mach. Intell..

[21] Ansari M.A., Crampton A., Garrard R., Cai B., Attallah M. (2022). A Convolutional Neural Network (CNN) classification to identify the presence of pores in powder bed fusion images. Int. J. Adv. Manuf. Technol..

[22] Abdelrahman M., Reutzel E.W., Nassar A.R., Starr T.L. (2017). Flaw detection in powder bed fusion using optical imaging. Addit. Manuf..

[23] Lin T.Y., Goyal P., Girshick R., He K., Dollár P. Focal loss for dense object detection. Proceedings of the IEEE International Conference on Computer Vision.

[24] Phan T.H., Yamamoto K. (2020). Resolving Class Imbalance in Object Detection with Weighted Cross Entropy Losses. https://arxiv.org/abs/2006.01413.

